# Using Thin Ultra-High-Molecular-Weight Polyethylene Coatings to Reduce Friction in Frost-Resistant Rubbers

**DOI:** 10.3390/polym16202870

**Published:** 2024-10-11

**Authors:** Elena Torskaya, Ivan Shkalei, Fedor Stepanov, Yulia Makhovskaya, Afanasy Dyakonov, Natalia Petrova

**Affiliations:** 1 Ishlinsky Institute for Problems in Mechanics RAS, 119526 Moscow, Russia; ioann_shiva@list.ru (I.S.); stepanov_ipm@mail.ru (F.S.); makhovskaya@mail.ru (Y.M.); 2 Department of Chemistry, North-Eastern Federal University, 677000 Yakutsk, Russia; afonya71185@mail.ru (A.D.); pnn2002@mail.ru (N.P.)

**Keywords:** UHMWPE coating, rubber, friction, contact problem, frictional heating

## Abstract

Frost-resistant rubbers retain their highly elastic properties over a wide temperature range. They are used in various friction units (e.g., seals), but their high friction coefficient and low wear resistance lead to the need for frequent replacement. In this paper, we propose applying thin (several hundred microns) UHMWPE coatings to formed rubber rings. The application technology depends on the required coating thickness. Friction tests of the coatings and pure UHMWPE were performed using the ball-on-disk (unidirectional sliding) scheme for various loads and velocities. In the experiments, the friction coefficients and temperatures near the contact area were determined. Friction tracks were studied using microscopy methods. The sliding contact of the ball and the two-layer material was modeled to obtain the dependences of the deformation component of friction on the sliding velocity for coatings of different thicknesses. UHMWPE is sensitive to frictional heating, so the thermal problem of determining the temperature in the contact area was also solved. It is shown that the minimum friction coefficient occurs for coatings with a thickness of 600 μm. At the same time, in the case of the 300 μm coating, the surface of the friction track is practically no different from the initial one. Thus, the studied combination of polymers provides antifrictional properties and wear resistance to the surface layer while maintaining the damping properties of rubber.

## 1. Introduction

Polymer composite materials (PCMs) are widely used in vehicles, quarry equipment, and process equipment since they have sufficiently high mechanical strength and processability, low density, corrosion resistance, and, in some cases, depending on the chemical nature of the selected polymers, antifriction properties, resistance in various environments, and frost resistance [1]. PCMs can be characterized as materials consisting of two or more phases separated by an interphase surface. To obtain them, polymers with different properties are used, including those belonging to different classes, such as thermoplastic and elastomer, in order to obtain a new set of properties [2,3]. Polymer phases are distributed differently in dispersed-filled, fibrous, and layered PCMs. A thin layer of polymeric material, which is applied to the surface of another material (substrate), can be considered as a coating [4,5]. Coatings have a variety of functional purposes, such as imparting high wear and abrasion resistance, and they have dielectric or electrically conductive properties while maintaining the properties of the volumetric matrix. This allows them to be used in sensors, bearings, and other rubber products (RPs), operating under complex loading conditions in extreme operating conditions [6,7]. In [8], the tribological characteristics of tantalum coatings sprayed onto a rubber substrate were studied. The use of such coatings leads to a significant improvement in the tribological characteristics of rubber (the friction coefficient decreases by an average of 1.7 times, and abrasive wear decreases by 1.8 times). The authors of [9] increased the wear resistance of elastomers by applying composite antifriction coatings. In [10], it is proposed to use the surface modification of silicone rubber by an electron beam and also aging to improve the service life of insulators. In [11], a titanium dioxide layer was successfully formed on a natural rubber substrate by the liquid-phase deposition method, which is expected to be used as an anti-aging layer for natural rubber.

Of considerable interest in the creation of PCMs is ultra-high-molecular-weight polyethylene (UHMWPE), which has a unique set of properties: high strength, wear resistance due to a low coefficient of friction, abrasion resistance, and high impact toughness, which is maintained at extremely low temperatures. Combining it with elastomeric materials that have high elasticity and damping properties gives interesting results [12,13,14].

As has been shown in a number of studies [15,16,17], an important mechanism of frictional interaction of UHMWPE with most materials is adhesion, which is caused by the high surface energy of the polymer.

The surface energy, in turn, depends significantly on temperature, so it is important to note the dependence of the tribological characteristics of UHMWPE on it. With an increasing temperature, the friction coefficient of UHMWPE increases [18]. Despite the fact that UHMWPE’s melting temperature is about 140 °C [15], and it is thermally stable up to 435 °C [19] (result of a thermal gravimetric analysis), during friction, a relatively small increase in temperature changes the frictional contact conditions of the material. In [20], it was shown that when UHMWPE slides against CoCr, an increase in the ambient temperature from 20 to 37 °C leads to a decrease in the friction coefficient and wear. On the contrary, in ballistic experiments on impact resistance, an increase in temperature to 80 °C does not have a significant effect on the deformation and destruction of UHMWPE laminates [21]. In addition, we should not forget about frictional heating. The contact temperature increases with increasing contact pressure and sliding velocity, which is shown in [22] for the friction of UHMWPE paired with ceramics. It is also noted that the gradient of temperature increase is greater at the beginning and gradually decreases over time. Thus, the effect of frictional heating on the tribological properties of the polymer must be taken into account.

Another friction mechanism important for the composite is energy dissipation due to the imperfect elasticity of the rubber. If the UHMWPE coating is thin, the deformation component can make a significant contribution to the total friction force. There are many experimental studies of rubber friction, particularly its deformation component. Pioneering papers [23,24,25] are those on rolling and sliding with lubrication. Many researchers have also developed models for studying hysteretic friction. In relation to the subject of this study, the most interesting subjects are spatial contact problems in quasi-static formulation for a slider and viscoelastic half-space [26,27,28,29]. The solutions are usually based on the results of sliding the concentrated force along the boundary of the viscoelastic half-space [30]. Problems were investigated in which the material model included a spectrum of relaxation times [26], which is more realistic for polymers. The influence of adhesive friction forces arising during sliding was investigated in [27,28]; it was found that for low compressible materials, the effect of friction on the contact problem solution is negligible. Inclusions in viscoelastic materials have an effect on energy dissipation in steady sliding contact [29]. The presence of a rigid coating on a viscoelastic body makes it necessary to complicate the model, which was carried out in [31].

The aim of this work is to study the features of dry friction of frost-resistant rubber with a UHMWPE coating using friction tests and modeling.

## 2. Materials and Experimental Methods

### 2.1. Materials

Two-layer composite material consists of ultra-high-molecular-weight polyethylene (GUR-4022, Ticona, Kelsterbach, Germany) as a coating and rubber as a substrate; nitrile butadiene rubber (a synthetic rubber derived from acrylonitrile and butadiene, NBR) obtained from SIBUR, Russia was used as the base. The composition contained all the necessary ingredients of rubber mixtures: rubber, filler (carbon black P-803), dispersant, and accelerator and activator of vulcanization. Sulfur was used as a vulcanizing agent.

The ingredients were mixed in a closed-type Plastograph EC Plus rubber mixer (Brabender, Duisburg, Germany). The initial temperature of the mixing rollers was 40 °C, the rotation speed was 25 rpm, the force was 25 N, and the total mixing time was 20 min. Vulcanization of the samples was performed on a GT-7014-H10C thermohydraulic press (Gotech, Taichung, Taiwan).

The production of molded rubber products by the compression method includes molding the rubber mixture from prefabricated blanks and their vulcanization at elevated temperature and pressure. The process temperature range is 150–180 °C. One of the methods for processing UHMWPE is hot pressing at an elevated temperature, and the temperature ranges for processing rubber and UHMWPE partially coincide, which allows samples to be obtained in one mold. At the vulcanization temperatures of the rubber mixture, UHMWPE powder begins to melt and create a high-quality coating on the surface of the elastomer. The coating application technology is described in sufficient detail in [13]. Ring samples with UHMWPE coating (600 μm and 300 μm) were obtained. The technology varied when manufacturing samples with different coating thicknesses. In the deposition of a 600 µm coating, UHMWPE powder was first molded (10 MPa for 5 min), combined with the rubber mixture, and held at 155 °C at 10 MPa for 20 min in a mold to vulcanize the elastomer and to press the thermoplastic, and then it was cooled in the same mold. For the thinner coating, the rubber mixture was first pre-vulcanized (155 °C × 10 MPa for 5 min), and then UHMWPE powder was applied (dry impregnation) and combined hot pressing was carried out with subsequent cooling in the mold. The total vulcanization duration was 20 min, including 5 min of pre-vulcanization of the rubber mixture and 15 min of post-vulcanization.

### 2.2. Methods for Determining Tensile Strength and Elongation

The tensile strength and relative elongation of the elastomer and UHMWPE samples were determined on an Autograph testing machine (Shimadzu, Kyoto, Japan) at room temperature in accordance with ISO 37:2024 [32]. Aggression resistance was determined using the test method for resistance (in unstressed state) to hydrocarbon environments by mass change according to ISO 1817:2024 [33]. AMG-10 oil was selected as the medium. The tests were performed at room temperature for 72 h. Differential scanning calorimetry (DSC, DSC 204 F1 Phoenix (Netzsch, Selb, Germany)) was used to determine the phase transition temperatures in the materials; the glass transition temperature was recorded.

For the successful operation of two-layer materials, it is necessary to ensure high bond strength between the layers. This was determined during their delamination. The idea of the method is to determine the force required to separate the UHMWPE layer from the rubber substrate. This was carried out using a testing machine for peeling off adhesive joints (Autograph (Shimadzu, Japan)), which allows the applied load (tensile force) to be perpendicular to the solid polymer substrate. The samples (double-sided blades) were prepared according to ISO 23529:2016 [34]. The crosshead speed was 500 mm/min. The experiments were performed at room temperature.

### 2.3. Friction Tests

Tribotester (MFT-5000, Rtec, San Jose, CA, USA) was used in the mode of unidirectional sliding. A steel ball (AISI 52100, diameter 10 mm) was loaded depending on the material: 10 N for bulk UHMWPE and 20 N for relatively thin and thick coatings. A lower load for the bulk material was chosen to minimize plastic deformations during friction [35]. The track radius was 23 mm. In an hour, the linear sliding velocity varied (all other parameters were equal) by increasing from 0.005 m/s to 1 m/s. This method was based on previous experiments with viscoelastic materials: the repeatability of the results is better when the contact pair does not open during the test [36,37]. At each velocity, the duration was 10 min. The dependence of the friction coefficient (COF) on time was recorded, as well as the temperature, which was measured at a distance from the contact zone by the radius of the ball and the thickness of the wall of its holder. The resulting values of the friction coefficient and temperature depending on the linear sliding velocity were obtained by averaging over several tests (not less than three).

It should be noted that the testing method is based on ASTM G99-23 [38], which was developed for testing coatings. The coating load is doubled in comparison to the standard. At the same time, the testing time is significantly shorter than that in ASTM G99-23. Wear was not assessed; only the ability of the composite to withstand the load without plastic deformations at the macro level and without cracks was tested. The absence of cracks and beads along the edges of the track, characteristic of plastic displacement of the material, was checked using optical microscopy methods.

## 3. Models

For better insights into the experimental results, a numerical model of sliding contact was developed. Modeling consists of two independent problems. The first one is the contact problem for an elastic sphere sliding on a viscoelastic half-space covered with a relatively hard coating. The second problem considers the steady process of heating occurring due to friction between the two bodies. The input and output parameters of the model are summarized in Table 1, and the mathematical formulations of the problems are shown below.

### 3.1. Contact Problem

We consider an elastic sphere (indenter) with a radius *R* sliding with a constant velocity *V* over the viscoelastic half-space covered with a relatively hard coating of thickness *H*. The sphere is loaded with vertical force *Q* and an unknown tangential force *Q_t_.* The elastic modulus and Poisson ratio of the considered materials are *E*^(*i*)^ and *ν*^(*i*)^, correspondingly, where index *i* = 0 is declared for the indenter, *i* = 1 for the coating, and *i* = 2 for the half-space. It is assumed that *E*_1_ *>> E*_2_.

The viscoelastic behavior of the half-space material can be expressed by substituting the elastic modulus $1/E_{2}$ in the Hook’s law with the Volterra integral operator $\hat{A}$:

(1) $$\hat{A}\left(\sigma (t)\right)=\frac{1}{E_{2}}\left[\sigma (t)+\int_{-\infty }^{t}\sigma (\tau )K(t-\tau )\;d\tau \right],\;K(t)=\sum_{i=1}^{N}k_{i}e^{-\frac{t}{\mu_{i}}}$$

where σ(t) is a time-dependent function, and 1/*k_i_* and µ*_i_* are the relaxation and retardation time spectrums, correspondingly.

A moving Cartesian coordinate system is used in the solution. Its *XY* plane corresponds with the undeformed top surface of the layer, the *OZ*-axis is the indenter axis of symmetry, and the *OX-*axis is co-directional to the vector of sliding.

The boundary conditions at the top surface of the layer are as follows (*z* = 0):

(2) $$\begin{array}{c} w\left(x,y\right)=f\left(x,y\right)+D,\;\left(x,y\right)\in \Omega \\ \sigma_{z}=0,\;\left(x,y\right)\notin \Omega \\ \tau_{xz}=0,\;\tau_{yz}=0 \end{array}$$

Here, *w* is the normal displacement of the top surface of the layer; *f* is the function defining the shape of the indenter; σ_z_, τ_xz_, and τ_yz_ are the normal and tangential stresses; Ω is the area of contact; and *D* is the slider penetration. We do not take into account tangential stresses in the contact for simplification, considering that they insignificantly change the normal pressure distribution.

The conditions at the layer–substrate interface (*z = H*) are as follows:

(3) $$w^{\left(1\right)}=w^{\left(2\right)},\;u_{x}^{\left(1\right)}=u_{x}^{\left(2\right)},\;u_{y}^{\left(1\right)}=u_{y}^{\left(2\right)}$$

Here, *u_x_* and *u_y_* are tangential displacements.

The equilibrium condition in the *OZ* direction is considered in the following form:

(4) $$Q=\iint_{\Omega }P\left(x,y\right)dxdy$$

The contact problem is solved using the Boundary Element Method. A rectangular mesh is built in an area exceeding the contact zone. Using a piecewise constant approximation, a linear system of equations is constructed based on the boundary conditions (2) and the equilibrium condition (4). The influence coefficients related to the normal displacement of the coated half-space are obtained using double integral Fourier transform (FT):

(5) $$\begin{array}{c} \kappa_{i}^{j}=-\frac{1}{G^{\left(1\right)}}\int_{0}^{\pi /2}\int_{0}^{\infty }\Delta \left(\lambda ,\varphi ,\gamma ,\chi \right)\cdot \cos\left(y_{ij}\gamma \sin\left(\varphi \right)\right)\cdot \\ \left[\cos\left(x_{ij}\gamma \cos\left(\varphi \right)\right)+\sum_{l}^{N}k_{l}\omega_{l}\frac{\omega_{l}V\gamma \cos\left(\varphi \right)\sin\left(x_{ij}\gamma \cos\left(\varphi \right)\right)+\cos\left(x_{ij}\gamma \cos\left(\varphi \right)\right)}{1+{\left(V\omega_{l}\gamma \cos\left(\varphi \right)\right)}^{2}}\right]d\varphi d\gamma \end{array}$$

*φ* and *γ* are the coordinates in the space of FT; χ = *E*^(1)^ (1 + ν^(2)^)/*E*^(2)^ (1 + ν^(1)^); Δ is a very cumbersome expression derived in [39] and linearly depends on pressure in the FT dimension. The derivation of the influence coefficients and solution of the mentioned linear system of equations is explicitly discussed in [31]. As a result, the contact pressure distribution *P_i_* and the penetration *D* are calculated. It is possible to calculate the deformation component of the friction force.

(6) $$\mu^{\left(d\right)}=\frac{\sum_{i=1}^{n}P_{i}\frac{\partial f(x_{i},y_{i})}{\partial x}}{Q}$$

### 3.2. Thermal Problem

When the contact problem is solved, it is possible to calculate the heating occurring due to friction both in the ball and in the polymer sample. The heating process is considered to be stationary. This leads us to the following equations for the coating (*i* = 1) and the half-space (*i* = 2):

(7) $$\Delta T^{\left(i\right)}=0,\;i=1,2$$

We will apply the DC-FFT algorithm [40] using the same mesh as in the contact problem in Section 3.1 to calculate the temperature. First, a frequency response function needs to be derived in the space of double FT.

Each *i* element of the mesh within the contact area Ω is subjected to a constant friction force moving with velocity *V* and therefore generating the specific power of frictional heat release:

(8) $$q_{i}=\mu VP_{i}$$

Let us consider that α < 1 is the part of the heat flux that is absorbed by the specimen and 1 − α is absorbed by the ball. At this point, α is unknown and is to be found later.

For a rectangular element on the surface with sides 2*a* and 2*b*, the heat flux is expressed in the following form (*z = 0*):

(9) $${\left.\frac{\partial T^{\left(1\right)}}{\partial z}\right|}_{z=0}=\left\{\begin{array}{c} -q(x,y)/\lambda^{\left(1\right)}\;\;\left|x\right|<a\wedge \left|y\right|<b\\ 0\;\;\;\;\;\;\left|x\right|>a\vee \left|y\right|>b \end{array}\right.$$

At the interface (*z = H*),

(10) $${\left.\lambda^{\left(1\right)}\frac{\partial T^{\left(1\right)}}{\partial z}\right|}_{z=H}={\left.\lambda^{\left(2\right)}\frac{\partial T^{\left(2\right)}}{\partial z}\right|}_{z=H},\;T^{\left(1\right)}(x,y,H)=T^{\left(2\right)}(x,y,H)$$

λ*_i_*, *i* = 1, 2 is the coefficient of thermal conductivity.

By applying double integral Fourier transform to the temperature *T*, Equation (7) can be reduced as follows:

(11) $$\frac{\partial^{2}{\tilde{T}}^{\left(i\right)}}{\partial z^{2}}-\gamma^{2}{\tilde{T}}^{\left(i\right)}=0\;(\gamma^{2}=\varphi^{2}+\beta^{2})$$

where ${\tilde{T}}^{\left(i\right)}\left(\varphi ,\beta ,z\right)$*—*the transformed temperature; *φ* and *β*—coordinates in FT. The boundary conditions (9) and (10) after the transformation are the following:

(12) $$\begin{array}{c} {\left.\frac{\partial {\tilde{T}}^{\left(1\right)}}{\partial z}\right|}_{z=0}=-\frac{\tilde{q}}{\lambda^{\left(1\right)}},\;{\left.\frac{\partial {\tilde{T}}^{\left(1\right)}}{\partial z}\right|}_{z=H}={\left.\frac{\partial {\tilde{T}}^{\left(2\right)}}{\partial z}\right|}_{z=H},\\ {\tilde{T}}^{\left(1\right)}(\varphi ,\beta ,H)={\tilde{T}}^{\left(2\right)}(\varphi ,\beta ,H),\;\tilde{q}=\frac{q}{\pi^{2}}\frac{\sin\left(a\varphi \right)\sin\left(b\beta \right)}{\varphi \beta } \end{array}$$

The general solution of Equation (11) is represented as follows [41]:

(13) $${\tilde{T}}^{\left(i\right)}(\gamma ,z)=A^{\left(i\right)}(\gamma )e^{-\gamma z}+B^{\left(i\right)}(\gamma )e^{\gamma z}$$

Using the boundary conditions (9) and (10), we obtain *A*^(*i*)^ and *B*^(*i*)^:

(14) $$\begin{array}{cc} A^{\left(1\right)}=\tilde{q}\frac{1}{\gamma (1+\omega e^{-2\gamma H})}, & B^{\left(1\right)}=\tilde{q}\frac{-\omega e^{-2\gamma H}}{\beta (1+\omega e^{-2\gamma H})},\\ A^{\left(2\right)}=\tilde{q}\frac{1+\omega }{\beta (1+\omega e^{-2\gamma H})}, & B^{\left(2\right)}=0,\;\;\omega =\frac{\lambda^{\left(2\right)}-\lambda^{\left(1\right)}}{\lambda^{\left(2\right)}+\lambda^{\left(1\right)}} \end{array}$$

Using expression (13) together with (14), we obtain the frequency response function and then use it in the DC-FFT algorithm [40] for calculating the temperature distribution at any depth *z* due to the applied α*q_i_.* In order to find the unknown coefficient α*,* we consider only one loaded element of the mesh. Knowing the coefficients of thermal conductivity of the contacting bodies, one can calculate the surface temperature in the element for the indenter *T_I_* and the sample *T_S_* depending on α, which is then obtained numerically from the following nonlinear equation:

(15) $$T_{I}\left(\alpha \right)-T_{S}\left(\alpha \right)=0,\;\left|\alpha \right|<1$$

All input and output parameters used in modelling are presented in Table 1.

The properties of UHMWPE were obtained from the nanoindentation test (elastic properties) and from [35,42] thermal conductivity. Rubber characterization was carried out by nanoDMA testing (see Appendix A).

## 4. Results

When selecting the materials to be combined, we proceeded from the need to ensure sufficient strength and elasticity of the two-layer material, its resistance to a hydrocarbon environment, and the possibility of its use at sufficiently low temperatures in Arctic conditions. UHMWPE and NBR fully comply with the selected criteria. This is confirmed by the data in Table 2, which show the properties of rubbers based on NBR and UHMWPE samples. When forming a two-layer material, the rate of structuring of the elastomer layer and the possibility of crosslinking at the rubber–thermoplastic phase interface are of great importance to ensure the integrity and monolithicity of the resulting products. Special attention was paid to the choice of vulcanizing system. The sulfur vulcanizing system is most often used for structuring rubbers. Varying the type of vulcanization accelerator allows for the rate and degree of elastomer vulcanization to be controlled and allows chemical crosslinking of NBR and UHMWPE. The following medium-activity accelerators were tested: mercaptobenzthiazole (MBT), which belongs to the group of thiazole-type accelerators; the amine-type accelerator diphenylguanidine (DPG); and an ultra-accelerator that provides an induction period—sulfenamide C—as well as their combinations with each other. For further studies, two systems were selected: sulfenamide C in combination with sulfur, zinc oxide, and a dispersant (sample 1) and a mixture of MBT + DPG with the same vulcanizing agent and activator (sample 2), since they provided the highest strength, the lowest degree of swelling, and sufficiently high frost resistance of the elastomer, as well as the highest adhesion between layers. The delamination force for the two-layer material obtained using sulfenamide C is 7.4 N/mm when using the MBT + DPG composition—7.3 N/mm—which is significantly higher than that for the other tested vulcanizing systems. In this case, the destruction is cohesion, i.e., in the process of delamination of the layers, destruction occurs along the elastomer, which indicates the formation of a high-quality and reliable connection between UHMWPE and the elastomer (the strength exceeds the cohesive strength of NBR). The glass transition temperature for the elastomer layer is in the range of −44.3–−45.4 °C; this indicator for UHMWPE could not be determined by the same method. According to published data [15], it is known that the glass transition temperature of UHMWPE is −120 °C.

A sulfur vulcanizing system with accelerators MBT + DPG (sample 2 in Table 2) was selected for the next step, which is tribological testing. When using this vulcanizing system, the rubber has higher strength values, slightly lower swelling values, and lower Tg values compared to another system (sample 1 in Table 2), i.e., it is more suitable for Arctic operating conditions.

**Figure 1:**
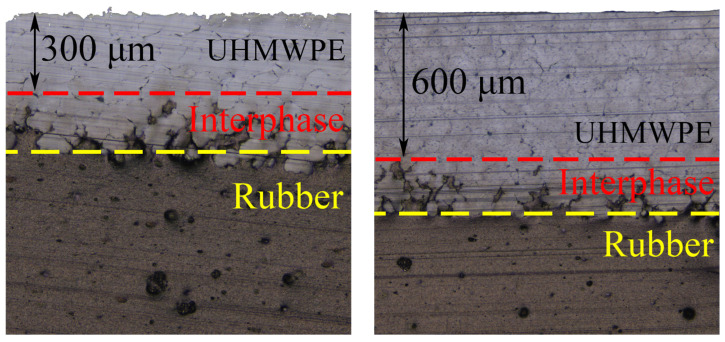
Optical images of cross sections of samples with thin and thick coatings.

On a noncontact optical profilometer S neox 3D (Sensofar-Tech, Barcelona, Spain), using a 20× objective, optical images of cross sections were obtained from which the actual coating thickness was determined (Figure 1).

**Figure 2:**
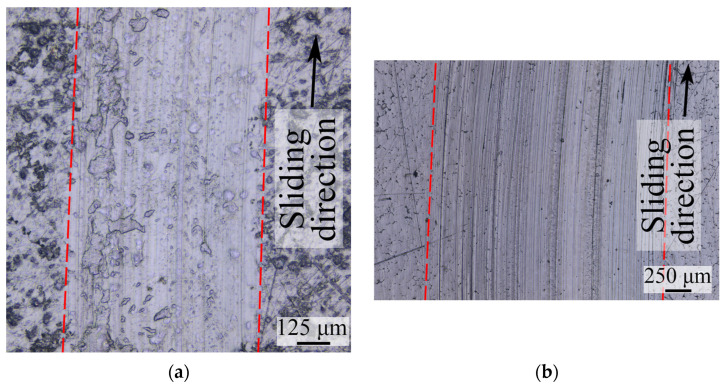
Optical images of friction tracks for bulk UHMWPE (**a**) and for composite with 600 µm UHMWPE coating (**b**).

The friction tracks formed during the full cycle of friction tests are shown in Figure 2. The friction track on the coating surface is wider than that on pure UHMWPE, which is explained by the significantly higher integral compliance of the composite. The surface structure of bulk UHMWPE and the coating differ due to differences in the formation technology. With the exception of abrasive plowing traces, the changes in the UHMWPE surface during friction are more noticeable for bulk UHMWPE. This is due to higher contact pressures. For thinner coatings, there are fewer abrasive traces, and the friction track boundaries are difficult to identify.

**Figure 3:**
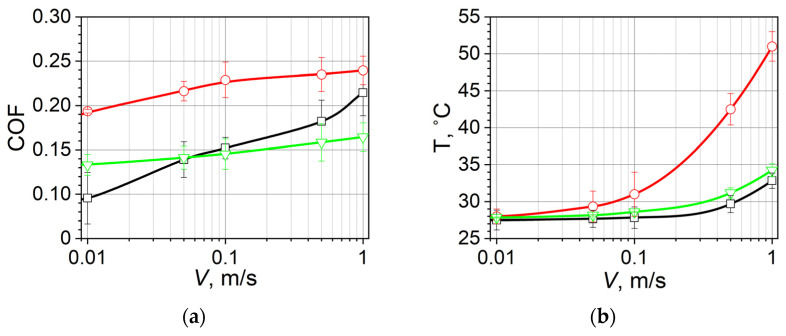
The dependence of the friction coefficient (COF) (**a**) and temperature (T) (**b**) on the sliding velocity *V* for bulk UHMWPE (black line, square), the 300 µm coating (red line, circle), and the 600 µm coating (green line, triangle).

The temperature and friction coefficient depend on the sliding velocity (Figure 3). It should be noted that the friction force in the case of bulk UHMWPE does not include the deformation component. Friction in a similar rubber without coating was studied earlier [37]; the friction coefficient was fixed in the range of 0.4–1.1 depending on the load and velocity parameters. The temperature was measured using a thermocouple (type K, tolerance class ±1.5 °C), located at some distance from the contact patch; therefore, it only allows for a qualitative assessment of the effect of velocity on heat flow going into the ball and caused by frictional heating.

**Figure 4:**
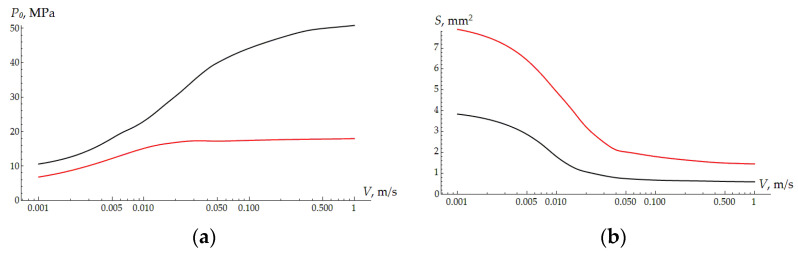
The maximum of the contact pressure *P*_0_ (**a**) and area of contact *S* (**b**) depending on the sliding velocity. H = 300 µm—red curve; H = 600 µm—black curve.

To analyze the friction mechanisms, it is necessary to consider the results of modeling the sliding contact. Figure 4 shows that the contact area size and the maximum contact pressure depend on the velocity for two coating thicknesses. The floating effect, characteristic of sliding over the surface of viscoelastic bodies, is expressed through a decrease in the contact area and an increase in the contact pressure. With an increase in velocity, these parameters tend to be constant due to the instantaneous modulus of elasticity of the viscoelastic material. The thicker the coating, the lower the integral compliance of the coating–substrate system, and the higher the maximum pressure. These parameters are directly related to the adhesive component of the friction force. The larger the contact area, the greater the adhesion [43]; therefore, for a thinner coating, adhesive friction should be more observable.

**Figure 5:**
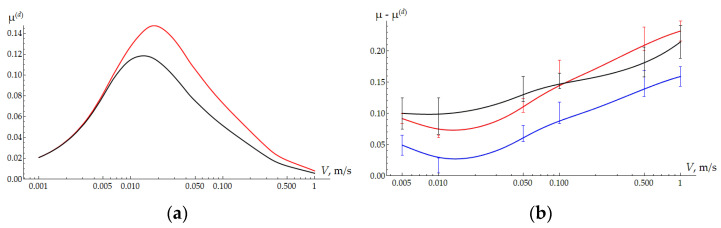
(**a**) The deformation component of the friction force depending on the sliding velocity. H = 300 µm—red curve; H = 600 µm—black curve. (**b**) The difference between the friction coefficient obtained in the experiment and the deformation component of the friction coefficient obtained from modeling. H = 300 µm—red curve; H = 600 µm—blue curve; clear UHMWPE—black curve.

The friction coefficient due to hysteresis losses in rubber also depends on velocity and the coating thickness (Figure 5a). As shown earlier [27,44], for weakly compressive materials, the effect of adhesive friction on deformation friction is negligible. As expected, hysteresis losses are greater for a thinner coating, especially at velocities at which the viscoelastic properties of the substrate are maximal. Subtracting the deformation component of the friction force from the total one recorded in the experiment allows us to analyze the adhesive component (Figure 5b). For samples with coatings at low velocities, the effect of frictional heating is negligible, while the effect of floating (reductions in the contact area and adhesion) is quite obvious. With an increase in velocity, heating leads to an increase in the surface energy of the polymer and, consequently, to an increase in adhesion.

**Figure 6:**
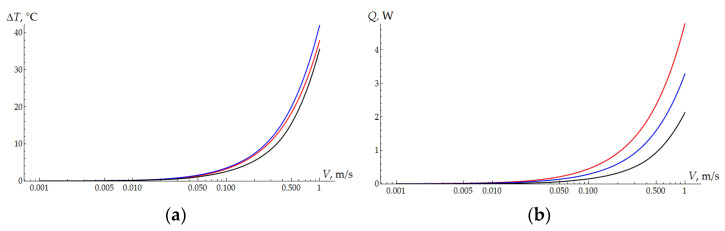
(**a**) The mean increase in the temperature within the contact area. H = 300 µm—red curve; H = 600 µm—blue curve; clear UHMWPE—black curve. (**b**) The heat flux depending on the sliding velocity. H = 300 µm—red curve; H = 600 µm—blue curve; clear UHMWPE—black curve.

As the results of the thermal problem solution show, the average temperature in the contact area is approximately the same for all three types of samples under consideration (Figure 6a). Due to the smaller contact area, the temperature of bulk UHMWPE is slightly higher, but in general, this explains the approximately identical behavior of the curves in Figure 5b. The difference in the curves obtained for the samples with coatings is explained by the difference in the contact area. Plastic deformation at the micro level also contributes to the total friction force, and it is greater the higher the contact pressure values. The difference in the surface topography of the coating and pure UHMWPE also affects the adhesion of the polymer. The curves of the heat flow into the ball as a function of velocity are shown in Figure 6b. The relative position of the curves coincides with the results in Figure 3b, although the experiments for the thick coating and bulk UHMWPE show similar results. The lowest flow in contact with bulk UHMWPE is explained by the half-lower load.

## 5. Conclusions

In conclusion, the combination of two polymers of different types allows us to obtain a damping material with a surface modification providing wear resistance and antifriction properties. The friction coefficient for the composite changes from 0.1 to 0.22, which is essentially smaller than the rubber COF (0.4–1.1). The disadvantage of UHMWPE is a low yield point, which, in combination with a high (for polymers) Young’s modulus, leads to the development of plastic deformation. The combination of thin layers of UHMWPE with rubber leads to decreases in contact and internal stresses, which allows us to avoid the demonstration of plasticity. The experimental results (dry friction) and modeling show that the friction coefficient depends on the coating thickness and sliding velocity. At the same time, the thicker the coating, the lower the damping capacity of the material. This means that for specific friction units and loading conditions, there is an optimal coating thickness, providing a combination of damping and antifriction properties.

## Figures and Tables

**Table 1:** The parameters used in modeling.

Input	*Q*, N	Vertical load	10; 20
	*R*, m	Radius of ball	0.005
	*E*^(0)^, MPa	Young modulus of ball	210,000
	ν^(0)^	Poisson ratio of ball	0.3
	*E*^(1)^, MPa	Young modulus of coating	1000
	ν^(1)^	Poisson ratio of coating	0.35
	*E*^(2)^, MPa	Young modulus of substrate	13.8
	ν^(2)^	Poisson ratio of substrate	0.45
	µ*_i_*, s	Spectrum of retardation times	0.13
	1/k*_i_*, s	Spectrum of relaxation times	1/40.14
	*H*, m	Coating thickness	0.0003; 0.0006
	λ^(0)^, W/(m·K)	Coefficient of thermal conductivity of indenter	50
	λ^(1)^, W/(m·K)	Coefficient of thermal conductivity of coating	0.4
	λ^(2)^, W/(m·K)	Coefficient of thermal conductivity of substrate	0.15
	µ	Friction coefficient	from the tests
	*V*, m/s	Sliding velocity	from the tests
Output	*P(x*,*y)*, Pa	Contact pressure distribution	
	*D*, m	Approach of indentor	
	µ^(d)^	Deformation component of friction coefficient	
	µ^(a)^	Adhesion component of friction coefficient	
	*T(x*,*y*,*z)*, °C	Temperature	

**Table 2:** Properties of rubber based on NBR and UHMWPE samples.

Properties	Tensile Strength, MPa,	Relative Elongation, %	The Degree of Swelling in Oil (AMG-10), %	Tg, °C
NBR, (sample 1)	11.7 ± 0.95	273 ± 21	15.5 ± 0.5	−44.3
NBR, (sample 2)	12.3 ± 0.8	238 ± 18	14.6 ± 0.4	−45.4
UHMWPE	37.4 ± 1.0	324 ± 24	0	−120 [15]

**Figure A1:**
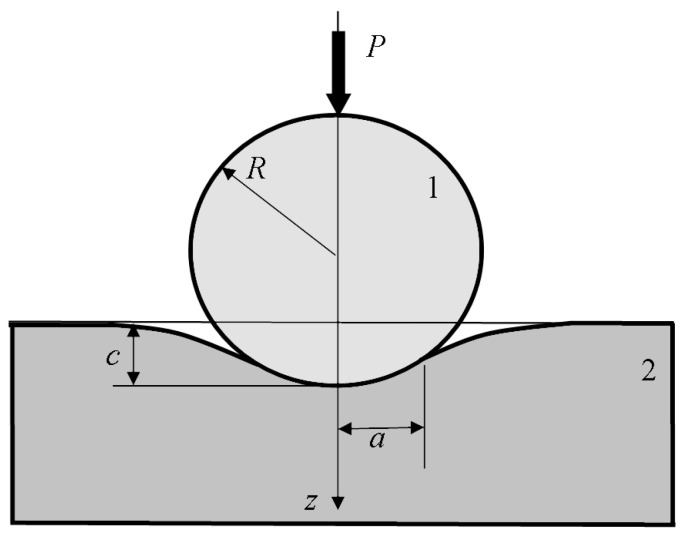
Scheme of contact in nanoDMA testing.

**Figure A2:**
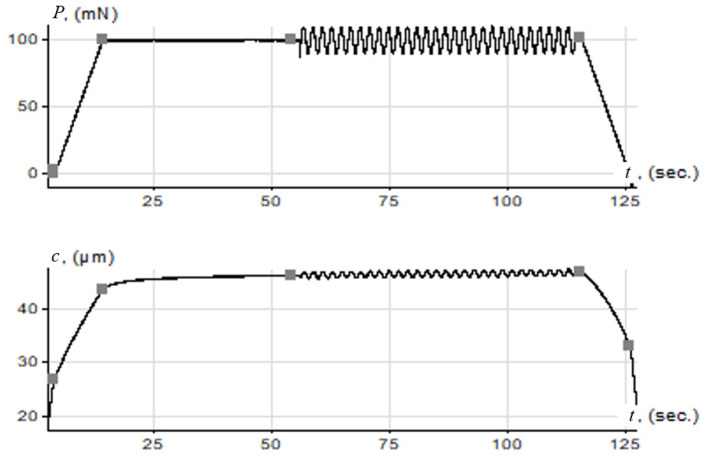
A typical diagram of a single nanoDMA test, including the regions of loading and unloading.

## Data Availability

The data are contained within the article.

## Appendix A

A scheme of contact during a typical nanoDMA test is presented in Figure A1. A rigid ball (1) is in contact with a rubber specimen (2) under the controlled normal force oscillating harmonically with time *t*:

(A1) $$P(t)=P_{0}+P_{s}\sin\omega t$$

It is assumed that in the regime of steady oscillations, the harmonic load (A1) makes the indentation depth *c* of the ball into the specimen vary harmonically with the same frequency ω but with a phase shift δ:

(A2) $$c(t)=c_{0}+c_{s}\sin(\omega t-\delta )$$

The contact radius *a* is a function of time as well, so the contact condition for the normal displacement w(r,t) of the specimen surface (*z =* 0) has the following form:

(A3) $$w(r,t)=c(t)-\frac{r^{2}}{2R},\;r\leq a(t)$$

where *r* is the radial coordinate of the cylindrical system of coordinates (r,z,θ), while the problem is axisymmetric with all functions independent of the angle θ.

The specimen is modeled by a linear viscoelastic half-space with the relaxation kernel presented as a sum of exponential terms, which, for a uniaxial loading, can be presented as follows [45]:

(A4) $$\sigma (t)=E_{1}\left[\varepsilon (t)-\int_{-\infty }^{t}\varepsilon (\tau )\Gamma (t-\tau )\;d\tau \right],\;\Gamma (t)=\sum_{i=1}^{N}B_{i}e^{-\frac{t}{T_{i}}}$$

where *E*_1_ is the instantaneous modulus of elasticity and Γ(t) is the exponential relaxation kernel. The reverse relationship through the creep kernel K(t) can be presented as follows:

(A5) $$\varepsilon (t)=\frac{1}{E_{1}}\left[\sigma (t)+\int_{-\infty }^{t}\sigma (\tau )K(t-\tau )\;d\tau \right],\;K(t)=\sum_{i=1}^{N}k_{i}e^{-\frac{t}{\mu_{i}}}$$

Here $k_{i}$ and $\mu_{i}$ are the parameters in the creep kernel (*i* = 1…*N*).

For the axisymmetric loading of the viscoelastic half-space with a constant Poisson’s ratio ν, from (A4) and the viscoelastic correspondence principle [45,46], it follows that the relation between the normal contact pressure *p* and displacement *w* has the following form:

(A6) $$w(r,t)=\frac{4}{\pi E_{1}^{*}}\int_{0}^{\infty }\;K\left(\frac{2\sqrt{rr'}}{r+r'}\right)\left(p(r^{\prime },t)+\int_{-\infty }^{t}p(r',\tau )\sum_{i=1}^{N}K_{i}e^{-\frac{t-\tau }{\mu_{i}}}d\tau \right)\frac{r^{\prime }dr^{\prime }}{r+r^{\prime }},$$

where $E_{1}^{*}=E_{1}/(1-\nu^{2})$ is the instantaneous reduced modulus of elasticity. The equilibrium condition has the following form:

(A7) $$P(t)=2\pi \int_{0}^{\infty }\;rp(r,t)dr$$

The inverse contact problem needs to be solved, which involves the determination of the instantaneous modulus $E_{1}^{*}$ and the parameters ($B_{i}$ and $T_{i}$) of the relaxation kernel and those ($k_{i}$ and $\mu_{i}$) of the creep kernel from the prescribed functions of harmonic loading (A1) and response (A2).

Following [47,48], we introduce the following functions:

(A8) $$q(x,t)=-2\int_{x}^{\infty }\frac{rp(r,t)}{\sqrt{r^{2}-x^{2}}}dr,\;u(x,t)=\frac{\partial }{\partial x}\int_{0}^{x}\frac{rw(r,t)}{\sqrt{x^{2}-r^{2}}}dr$$

Relation (A8) specifies an Abel-type transformation which reduces Equation (A6) to the equivalent form [49,50]:

(A9) $$q(x,t)=E_{1}^{*}\left[u(x,t)-\int_{-\infty }^{t}u(x,\tau )\sum_{i=1}^{N}B_{i}e^{-\frac{t-\tau }{T_{i}}}\;d\tau \right]$$

whereas the contact condition (A3) accounting for (A2) takes the following form:

(A10) $$u(x,t)=c_{0}+c_{s}\sin(\omega t-\delta )-\frac{x^{2}}{R},\;x\leq a(t)$$

By substituting the contact condition (A10) into Equation (A9) and dropping a decaying exponential term, one can obtain the following expression for the function *q*:

(A11) $$q(x,t)=E^{*}\left(c_{0}-\frac{x^{2}}{R}\right)+E^{\prime }c_{s}\sin(\omega t-\delta )+E^{\prime \prime }c_{s}\cos(\omega t-\delta )$$

where $E^{*}$ is the relaxed modulus of elasticity, while $E^{\prime }$ and E″ are the storage and loss moduli, respectively, which are specified by the following expressions:

(A12) $$E^{*}=\frac{E_{1}^{*}}{\alpha },\;E^{\prime }=\sum_{i=1}^{N}E_{i}\frac{\alpha T_{i}^{2}\omega^{2}+1}{T_{i}^{2}\omega^{2}+1},\;E^{\prime \prime }=\sum_{i=1}^{N}E_{i}\frac{\omega T_{i}(\alpha -1)}{T_{i}^{2}\omega^{2}+1}$$

where the values α and $E_{i}$ (i=1…N) are introduced so that the relations are satisfied as follows:

(A13) $$B_{i}=\frac{(\alpha -1)E_{i}}{E_{1}^{*}T_{i}},\;\;i=1\ldots N,\;E^{*}=\sum_{i=1}^{N}E_{i}$$

The obtained expression (A11) for the function *q* satisfies Equation (A9) only for the points of contact area which always stay in contact with the ball, i.e., for $x\leq a_{\min}$, where $a_{\min}\leq a(t)\leq a_{\max}$ is the range of variation of the contact radius during the cyclic indentation. Under the condition$c_{s}\ll c_{0}$, we can assume that expression (A11) is approximately satisfied for all points of the contact area. Then, from the continuity condition q(a,t)=0, we obtain the approximate expression for the contact radius evolution:

(A14) $$a(t)=\sqrt{Rc_{0}}{\left(1+\frac{c_{s}}{c_{0}}H(t)\right)}^{1/2},\;$$

where

(A15) $$H(t)=\frac{1}{E^{*}}\left[E^{\prime }\sin(\omega t-\delta )+E^{\prime \prime }\cos(\omega t-\delta )\right]$$

The contact pressure *p(r,t)* is determined from the inverse transformation to (A8) [50]:

(A16) $$p(r,t)=-\frac{1}{\pi }\int_{r}^{a(t)}\frac{1}{\sqrt{x^{2}-r^{2}}}\frac{\partial q(x,t)}{\partial x}dr$$

By substituting (A11) with (A14), by putting (A15) into (A16), taking the integral, and substituting the result into the equilibrium condition (A7), we obtain the following for the load:

(A17) $$P(t)=\frac{4E^{*}}{3R}\sqrt{Rc_{0}^{3}}{\left(1+\frac{c_{s}}{c_{0}}H(t)\right)}^{3/2}\approx \frac{4E^{*}}{3R}\sqrt{Rc_{0}^{3}}\left(1+\frac{3}{2}\frac{c_{s}}{c_{0}}H(t)\right)$$

**Experimental setup.** Measurements were carried out on the NanoScan-4D hardness tester (TISNUM, Moscow, Russia). The design of the device makes it possible to set the load with an accuracy of about 100 μN in the range of up to 3.5 N. The displacement is measured with an accuracy of about 3 nm in the range of up to 200 μm.

The NBR specimen was tested at room temperature (*T* = 22 ± 3 °C). To avoid the Patrikeev–Mullins effect, the specimen surface was preliminarily indented with a maximum frequency of 60 Hz for 60 s, and then the surface was left alone for 20 s, and after that, the surface was indented again at the same place with a chosen frequency. The relaxation of each specimen before the next measurement lasted 40 s, and the oscillations lasted 60 s.

An Al_2_O_3_ ball with a radius *R* of 2 mm was used as an indenter. The load was set in accordance with the harmonic law (A1), where *P*_0_ = 100 mN, *P_s_* = 10 mN, and ω = 2π*f*. A typical time diagram of a nanoDMA test with the controlled load *P* and registered depth *c* variating in time is shown in Figure A2.

Tests were performed at six frequencies *f_j_* (j=1…n, n=6). For each frequency, the corresponding mean depth $c_{0j}$ and amplitude depth $c_{sj}$ of the ball indentation were registered, as well as the phase difference $\delta_{j}$ between the load and depth oscillations. The results of the measurements are presented in Table A1.

**Table A1:** The results of the nanoDMA test for an NBR specimen.

j	$f_{j}$, Hz	$c_{0j}$, nm	$c_{sj}$, nm	$\delta_{j}$, rad
1	0.1	87,493	7033	0.138
2	0.2	85,832	6993	0.126
3	0.5	84,761	6317	0.044
4	1	83,525	6018	0.038
5	6	83,607	5333	0.057
6	30	83,607	6700	0.161

**Numerical algorithm.** To obtain the expressions for the numerical processing of the testing results, the approximate solution for the load *P* on the right-hand side of (A17) is compared with the exact form (A1). In these relations, by setting the terms containing cosωt equal to zero and equating the terms containing sinωt and the free terms, we obtain the expressions for the storage ${E^{\prime }}_{j}$ and loss $E_{j}^{\prime \prime }$ moduli and a relaxed elastic modulus for a *j*-th frequency:

(A18) $$E_{j}^{\prime }=\frac{P_{s}\cos\delta_{j}}{2c_{sj}\sqrt{Rc_{0j}}},\;E_{j}^{\prime \prime }=\frac{P_{s}\sin\delta_{j}}{2c_{sj}\sqrt{Rc_{0j}}},\;E_{j}^{*}=\frac{3P_{0}}{4c_{0j}^{3/2}R^{1/2}}$$

The first two relations of (A18) coincide with the formulas for the storage and loss moduli obtained in (A18), which were obtained in [51], while the third relation coincides with the Hertz formula [47]. Taking into account Equation (A12), from (A18), we obtain the following system of equations (j=1…n):

(A19) $$\sum_{i=1}^{N}E_{i}\frac{\alpha T_{i}^{2}\omega_{j}^{2}+1}{T_{i}^{2}\omega_{j}^{2}+1}=E_{j}^{\prime },\;\sum_{i=1}^{N}E_{i}\frac{\omega_{j}T_{i}(\alpha -1)}{T_{i}^{2}\omega_{j}^{2}+1}=E_{j}^{\prime \prime },\;\sum_{i=1}^{N}E_{i}=E^{*}\;$$

where $\omega_{j}=2\pi f_{j}$. System (A19) is to be solved for the unknown constants (*E_i_*, *T_i_*, and α) of the relaxation spectrum (i=1…N). To make solving this nonlinear system easier, one can set values of *T_i_*, α, and *E** and then solve the obtained linear system for a set of *E_i_* [51].

To set the values of *T_i_*, α, and *E**, we use the solution obtained in the case of *N =* 1. In this case, the system of equations (A19) with (A18) is solved analytically to obtain the values *T_j_* and α*_j_* for each *j*-th frequency:

(A20) $$T_{j}=\frac{1}{\omega_{j}}\sqrt{\frac{P_{s}^{2}-F_{j}^{2}}{F^{2}\alpha_{j}^{2}-P_{s}^{2}}}\;$$

(A21) $$\alpha_{j}=\frac{P_{s}}{F_{j}}\frac{P_{s}F_{j}\sin^{2}\delta_{j}+(P_{s}^{2}-F_{j}^{2})\cos\delta_{j}}{P_{s}^{2}\cos^{2}\delta_{j}-F_{j}^{2}}$$

where $F_{j}=2E_{j}^{*}\sqrt{Rc_{0j}}c_{sj}$. We set the values of α and $E^{*}$ as averaged over j=1…n:

(A22) $$\alpha =\frac{1}{n}\sum_{j=1}^{n}\alpha_{j},\;E^{*}=\frac{1}{n}\sum_{j=1}^{n}E_{j}^{*},$$

while the relaxation times *T_j_* are evaluated by (A20). We set the number of relaxation times in the spectrum (*N* = 2) and take the smallest and the largest values of those calculated by (A20) as *T_j_*.

Then, Equation (A19) specifies an overdetermined linear system (*n* = 6; *N* = 2) for a set of moduli *E_i_* which is solved by the least squares method. After this, the instantaneous elastic modulus is calculated as $E_{1}^{*}=\alpha E^{*}$, while the parameters *B_i_* of the relaxation kernel Γ(t) (A4) are calculated from (A13).

After calculating the parameters *B_i_* and *T_i_* of the relaxation kernel Γ(t) (A4), one can determine the parameters *k_i_* and *µ_i_* of the creep kernel *K*(*t*) (A5). It is shown that the parameters of the exponential creep and relaxation kernels are related as follows [45]:

(A23) $$1+\sum_{i=1}^{N}\frac{k_{i}}{\mu_{i}^{-1}-T_{s}^{-1}}=0,\;1+\sum_{s=1}^{N}\frac{B_{s}}{\mu_{i}^{-1}-T_{s}^{-1}}=0,\;i,s=1\ldots N$$

The equations shown in (A23) are an algebraic system to be solved for *k_i_* and µ*_i_*. For *N* = 2, this system is reduced to a quadratic equation and is solved analytically. The parameters of the relaxation *B_i_*, *T_i_*, and creep *k_i_* and µ*_i_* kernels, as well as the instantaneous modulus $E_{1}^{*}$ and ratio α of retardation to relaxation times, which are calculated based on the results from Table 1, are presented in Table A2.

**Table A2:** The parameters of the relaxation and creep kernels for an NBR specimen.

i	$T_{i}$, s	$B_{i}$	$\mu_{i}$, s	$k_{i}$	$E_{1}^{*}$, MPa	α
1	4.77	0.14	0.030	5.54	2.76	5.03
2	0.025	5.59	20.67	0.18		
